# Drug pricing and reimbursement information management: processes and decision making in the global economy

**DOI:** 10.1080/20016689.2017.1340747

**Published:** 2017-06-30

**Authors:** Dimitrios Tsourougiannis

**Affiliations:** ^a^ Medical Affairs, HEOR, EMEA Astellas Pharma Europe Ltd, Chertsey, UK

**Keywords:** Pharmaceutical business environment, market access, external reference pricing, e-pricing system

## Abstract

**Background**: Cost-containment initiatives are re-shaping the pharmaceutical business environment and affecting market access as well as pricing and reimbursement decisions. Effective price management procedures are too complex to accomplish manually. Prior to February 2013, price management within Astellas Pharma Europe Ltd was done manually using an Excel database. The system was labour intensive, slow to update, and prone to error. An innovative web-based pricing information management system was developed to address the shortcomings of the previous system.

**Development**: A secure web-based system for submitting, reviewing and approving pricing requests was designed to: track all pricing applications and approval status; update approved pricing information automatically; provide fixed and customizable reports of pricing information; collect pricing and reimbursement rules from each country; validate pricing and reimbursement rules monthly. Several sequential phases of development emphasized planning, time schedules, target dates, budgets and implementation of the entire system. A test system was used to pilot the electronic (e)-pricing system with three affiliates (four users) in February 2013.

**Outcomes**: The web-based system was introduced in March 2013, currently has about 227 active users globally and comprises more than 1000 presentations of 150 products. The overall benefits of switching from a manual to an e-pricing system were immediate and highly visible in terms of efficiency, transparency, reliability and compliance.

**Conclusions**: The e-pricing system has improved the efficiency, reliability, compliance, transparency and ease of access to multinational drug pricing and approval information.

## Introduction

Pharmaceutical companies aim to provide safe and efficacious treatments for patients whilst ensuring a predictable business model; to do this they operate within an environment that tries to balance three broad objectives: to make medicines accessible and affordable to patients; to control public spending; and to provide incentives for innovation [1]. These factors are often conflicting from an economic perspective; for example, whilst most stakeholders welcome innovation in the pharmaceutical industry, it comes at the expense of increasing costs of medicines and other healthcare-related expenditures. Similarly, the balance between financially viable industry business models and patient access to affordable medicines continues to challenge pharmaceutical companies and policy makers alike.

**Figure 1.**
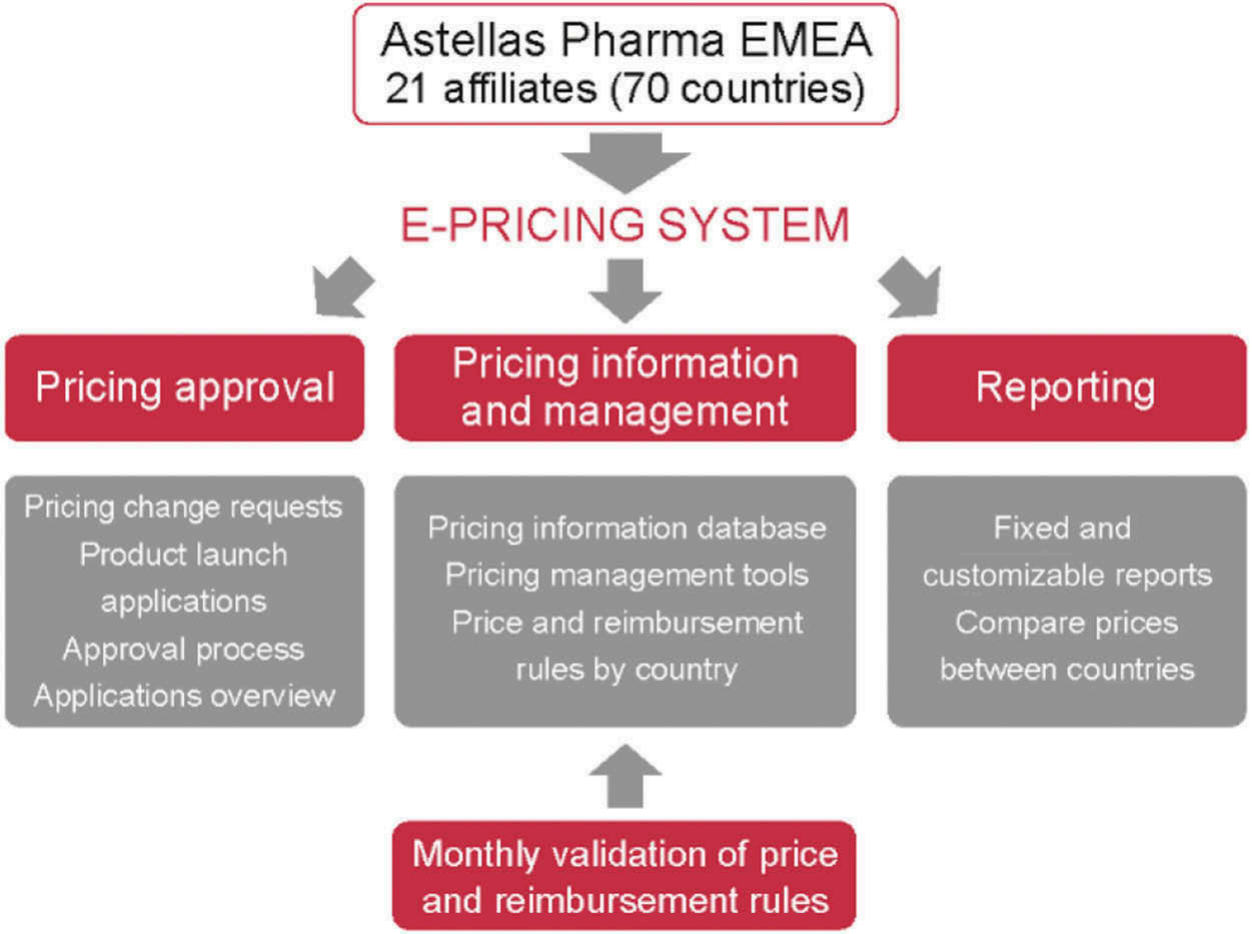
Planned spread of process to be incorporated within the Astellas e-pricing system. The new e-pricing system aimed to support pre-existing product, new presentation of pre-existing product and new product pricing processes within Astellas Pharma EMEA 21 afﬁliates (70 countries).

**Figure 2.**
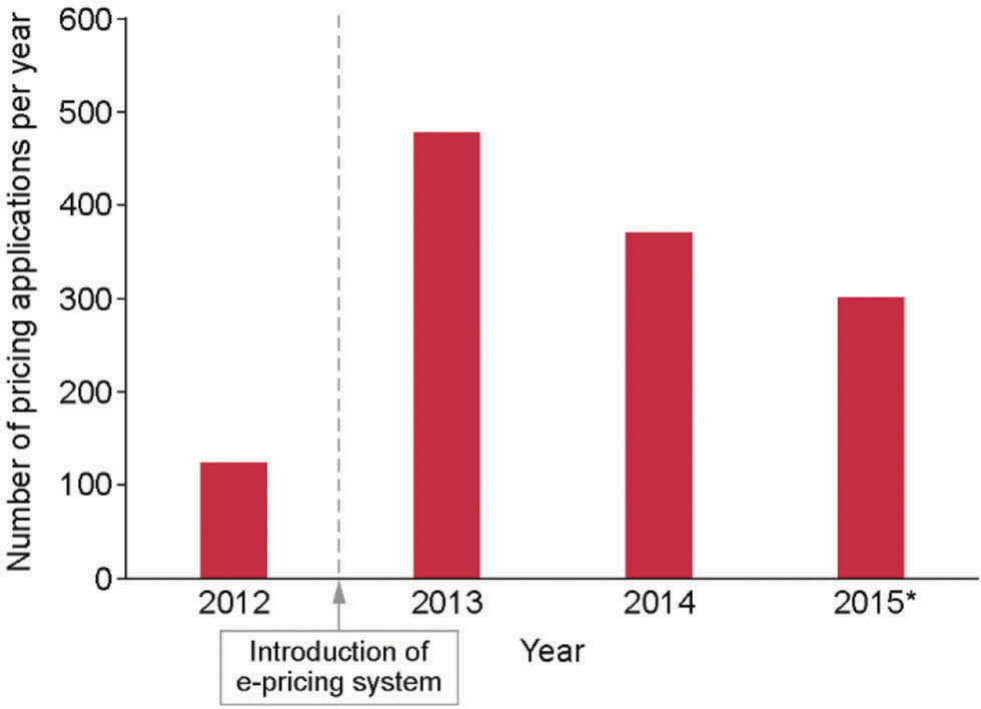
Number of pricing applications before and after the introduction of the e-pricing system. In 2012, the final year of the Excel-based system, there were 124 pricing applications. Since the introduction of the e-pricing system more than 300 applications have been processed every year. *Up to September 3, 2015.

**Figure 3.**
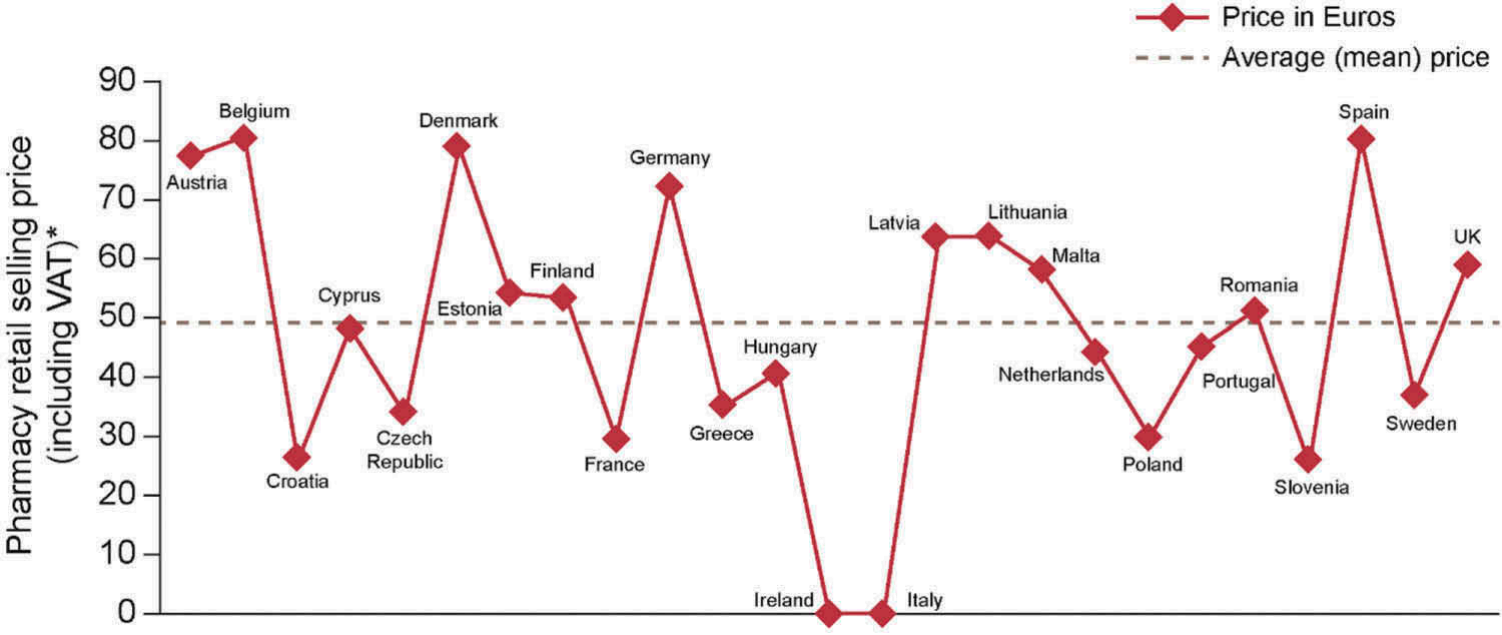
A typical price comparison chart for a product in 25 European Commission countries. The e-pricing system has improved price visibility; reporting functionality provides users with live access to pricing levels and pricing across the company. *No price recorded means the pharmacy retail price (including VAT) was not documented in the e-pricing system for that country.

The pharmaceutical industry continues to play a major role in the world economy. In 2015, worldwide prescription drug sales based on the top 500 pharmaceutical and biotech companies were $742 billion; sales forecasts estimate the pharmaceutical industry is set to grow at 6.3% per year from 2016 reaching $1.12 trillion by 2022 [2]. Closely related to this growth are concerns in many countries about rising expenditure in healthcare and the associated debate relating to the pricing of medicines. Consequently, this is a period marked by considerable challenges for the global pharmaceutical industry as it faces burgeoning cost containment initiatives by payers, governments and healthcare insurance organizations.

The availability and price of a pharmaceutical product often differs between countries to reflect each country’s valuation of the product’s health benefit (as determined by the local needs of national and regional healthcare providers), approval and market access regulations, and income levels [3]. These cost containment initiatives have many different approaches, with detailed rules and practices [3]. For the pharmaceutical companies, these cost-containment initiatives are re-shaping the business environment and affecting market access as well as pricing and reimbursement decisions that are important in determining the likely commercial success of a pharmaceutical product in the marketplace. This article examines internal and external pricing challenges and related business processes within Astellas Pharma  EMEA (Europe, the Middle East and Africa), as well as reporting the development of a bespoke online pricing and reimbursement tool.

### Situation analysis: external factors

A broad range of strategies, with different goals and effects, are used in an attempt to contain overall healthcare costs such as co-payment by patients, risk sharing agreements, and non-coverage by health plans or within the reimbursement system. The control of pharmaceutical drug prices forms an important part of cost-containment strategies and may include direct and indirect price controls, profit controls, reference pricing, physician budget constraints, prescribing guidelines, marketing approvals, and limits on promotion [3–5]. Reference pricing of medicines is an increasingly popular cost-control strategy. Reference pricing can be used to set prices either based on local prices of reference drugs in the same therapeutic class (internal referencing) or based on the prices of reference drugs in other countries (external referencing) [5]. The WHO defines external reference pricing (ERP; or international reference pricing) as, ‘The practice of using the price(s) of a pharmaceutical product in one or several countries in order to derive a benchmark or reference price for the purposes of setting or negotiating the price of the product in a given country’ [5]. The European Commission uses a similar definition [6].

ERP is widely used by policy makers in both the European Union (EU) and in non-EU countries to decide the prices of pharmaceutical products in their territories,[4–9] but there are numerous variations in the schemes adopted. For example, the ‘basket’ of reference countries may include a few neighbouring countries with a similar standard of living, or numerous countries from around the world. In addition, the reference price may be set using the median or mean price or the lowest price in the basket [4,5].

The market created by cost containment measures results in a complex framework for pharmaceutical price optimization. Price optimization describes the process whereby pharmaceutical manufacturers seek to optimize the returns from any given pharmaceutical product, thereby creating investment for future research into innovative technologies aimed to benefit patients. This is a highly complex undertaking that requires the use of effective price management procedures to model accurately the financial impact of various pricing levels for any given product. Indeed, it would appear that the management of pricing structures with many variables is too complex to accomplish manually and is a significant cost.

### Situation analysis: internal factors

Pharmaceutical companies are estimated to spend about 4% of cost of goods sold on pricing reconciliation processes and procedures [10]. However, according to an Accenture survey,[11] although 80% of the executives surveyed stated that pricing optimization is one of the top three strategic priorities for their companies, two-thirds of pharmaceutical companies do not have sophisticated pricing capabilities. In another survey, 61% of respondents from global pharmaceutical manufacturers highlighted a critical need for a centralized database providing timely, accurate pricing information, and 84% demanded better analytical capabilities to understand the potential impact of pricing actions [12].

Prior to March 2013, as in many pharmaceutical companies, pricing information management within Astellas Pharma EMEA relied on manual processes, an Excel-based database, spreadsheets, email and a formal approval system to improve governance and minimize pricing errors. This manual process was not considered to be robust as it was vulnerable to errors and inconsistencies, and was conducted within a limited technological support framework.

Typically, pricing applications were prepared by the EMEA affiliate countries using Excel spreadsheet documents that were then sent by email for processing and approval. Within Astellas Pharma EMEA, changes to product prices by each affiliate required review by the Commercial Operations team followed by approval from the Pricing Director and a Senior Vice-President. The Excel-based database was updated manually once a month to incorporate recent pricing approvals retrospectively and a number of dashboards were updated on an ad hoc basis. Furthermore, no ERP modelling tool was used at that time; the pricing and reimbursement rules for each country, knowledge of which is necessary for the price reference calculations, were collected from affiliates annually and updated using Excel.

A number of problems were identified with the use of this manual pricing information management system. An inherent time lag was produced by the requirement for forms to be printed, signed, scanned and emailed to the next approver. Additionally, updates were slow to appear on Excel reports, since they needed to be developed manually, resulting in the potential for the prices and rules to be outdated or invalid by the time the information was disseminated to all stakeholders. Furthermore, the process of manually entering the approved data into the Excel-based system introduced a high potential for human error, which could result in decisions being made using erroneous data. In addition, the process was labour intensive; re-typing data required time, resources, and strict attention to detail. The project team estimated that over one year, several weeks of middle and senior managers’ time were used in the preparation and processing of all the requested information. The net effect of these factors translated into significant direct and indirect negative effects on revenue and costs for the company. The changing internal and external business environment presented a unique opportunity to drive for investment to improve global pricing capabilities. It was also apparent that the development of an electronic system capable of managing pricing information quickly and accurately would be crucial to the implementation of effective pricing management strategies and controls.

### Addressing the problem

A project team was formed within Astellas Pharma EMEA, led by the author and comprising key stakeholders. This project team defined and implemented an innovative web-based electronic (e)-pricing and reimbursement information management system (the Astellas’ e-Pricing System) to better manage the process for approving price changes and to address the shortcomings of the previous Excel-based system. The overall aims of the e-Pricing system were to reduce the time and effort required to approve price change requests and to minimize the risk of using incorrect or unapproved prices. Simultaneously, it was envisaged that the e-pricing system would increase the visibility of pricing data to affiliates and improve compliance with the pricing approval process. The project team planned to develop a secure web-based system (intranet) for submitting, reviewing and approving pricing requests, initially for use by 21 affiliates in more than 70 countries in EMEA. The system would include all products sold by Astellas Pharma EMEA.

### Developing the solution

The e-pricing project team established clear roles and responsibilities and worked together throughout the project to establish the rationale and business requirements, and to design and then implement the system. A sequential development approach (a modified waterfall model as described by William Royce in 1970) was used [13]. Development occurred steadily through several sequential phases with an emphasis on planning, time schedules, target dates, budgets and implementation of the entire system [14,15]. Tight control was maintained over the life of the project through written documentation, formal reviews, and approval/signoff by the user and information technology management occurring at the end of most phases before beginning the next phase. An iterative approach was employed, in accordance with the waterfall model; a prototype was created and tested, and any new information gained was incorporated prior to delivery of the live system.

The first phase of the project was to establish the business case and achieve leadership buy-in. Once this had been achieved, a comprehensive stakeholder requirements analysis was conducted involving a thorough assessment of the original price processes and internal interviews with key personnel (senior managers from the company’s Sales, Marketing and Commercial teams). System design began with a definition of technical requirements (incorporating a requirement matrix – mapping user requirements to functions) and the preparation and approval of a functional specification to include all required elements (Table 1).

**Table 1.** Stakeholder requirements included in the functional specification for the e-pricing system.

Enable all applications for price changes to be submitted electronically.
Track all pricing applications and their stage of approval.
Update approved pricing information automatically.
Provide fixed and customizable reports of pricing information.
Collect pricing and reimbursement rules from each country.
Validate pricing and reimbursement rules monthly to ensure that information is up to date.
Provide different access levels within the system to ensure appropriate use.
Provide diverse reporting functionality.

#### System building and testing

The three processes the system planned to support are shown in Figure 1 (pre-existing product, new presentation of pre-existing product and new product). System building and testing occurred in parallel. This approach enabled the concurrent development of governance, support and communications plans. A software maintenance protocol was also prepared during the software development phase.

The chosen browser platform was Microsoft Internet Explorer 8.0. Ten access levels were planned within the system: Viewer, HQ Viewer, Applicant, General Manager, Area General Manager, Technical Approver, Director of Pricing, Finance Director, Head of Marketing and Senior Vice-President. Secure access was to be controlled using Windows Authentication to compare each user’s Windows login with details for valid users of the system, and then allow registered users to navigate to any of the permissible pages in the system.

#### Piloting

To ensure the final system was going to meet requirements and be well-adopted, a test system was used to pilot the (e)-pricing system with three affiliates (four users) in February 2013. This pilot e-pricing system provided an early sighting of the ‘look and feel’ of the software and provided valuable feedback from affiliates that the project team used to enhance the appearance and content prior to going live. This feedback resulted in several iterations of the e-pricing system and helped produce a final version of the system that is effective and intuitive.

#### Training and roll-out

The online e-pricing system was planned to be first introduced to Astellas Pharma EMEA and its affiliates. Care was taken to involve affiliate representatives early and explain the impact the new solution would have on their activities. Support procedures for the e-pricing system were developed, including training materials and an operations manual. A ‘Super User’ was assigned to each affiliate, who was trained by a Senior Manager from Astellas Pharma EMEA, and who was then responsible for training any additional users in their affiliate, handling technical problems or referring them to an IT helpdesk, as appropriate.

### Outcomes

The adoption of an integrated team approach and a collaborative working contributed to the delivery of a user-friendly, robust, and scalable e-pricing system to support the need for a new price management approach. Collaborative working was particularly beneficial during the final testing of the software. All team members supported testing, training and problem resolution, thus enabling the Astellas e-pricing system to go live earlier than planned.

The e-pricing system was successfully rolled-out to all Astellas Pharma EMEA affiliates concurrently in March 2013; it was upgraded with the pricing and reimbursement rules collection component in February 2014. The system currently has about 227 active users globally and comprises more than 1000 presentations of 150 products with various doses and formulations.

The overall benefits of switching from a manual system to e-pricing were immediate and highly visible in terms of efficiency, transparency, reliability and compliance.

#### Efficiency

Efficiency benefits are highlighted in Table 2. There has been a marked reduction in the time required for approval of pricing requests from approximately 40 days with the Excel-based system to 6.5 days with the e-pricing system. Pricing information, which previously required a formal request and several days to process, is now updated live online and is available to users instantly. The estimated time needed to create monthly updates has also decreased from 1–2 days to 1–2 minutes (for simple reports) and complicated analyses that would previously have been outsourced can be performed internally.

**Table 2.** Excel-based versus e-pricing system: Time required to perform various pricing-related and reporting functions.

	Pricing system	
Estimated time to complete tasks	Excel-based	e-Pricing
Pricing approval/rejection	40* days	6.5* days
Respond to internal use pricing information request	1–4 days	<1^‡^ day
Respond to P&R rules information request	1–2 weeks	<1^‡^ day
Regulatory use pricing information request	1–2 weeks	1–2 days
Create monthly reports	1–2 days	1–2^‡^ minutes
Validate price and P&R rules	1–3 weeks	<1^‡^ day

*Mean time; ^‡^Information available on system (live update). P&R = pricing and reimbursement.

Furthermore, the number of pricing applications has increased from 124 in 2012, with the original Excel-based system, to more than 300 applications every year since 2013 (335 applications were fully processed in 2014 and 307 in 2015; Figure 2).

#### Transparency

The e-pricing system has improved price visibility and the available information is transparent, for the first time, to all stakeholders across the company. The built-in reporting functionality provides live access to pricing levels and pricing comparisons for all products and affiliates/countries (see Figure 3 for an example). Applicants and Approvers have an overview of all applications in the system, and can see at a glance the approval status and an estimation of when the prices are to become effective if approved.

Transparency and access to all users of price information make it easy to exchange information and make decisions promoting optimal pricing, thus ensuring that products reach patients at a cost that is fair, and which retains the necessary stimulus for continued investment in innovative therapies.

#### Reliability and compliance

The new system delivers a reliable solution for the end user that saves time and is user friendly. The system has received positive feedback from users and, because of its ease of use, constantly updated content and robust validation function to prevent the use of erroneous pricing information, the system has a very high degree of compliance within the organization.

### Ongoing roll-out and development

Robust governance and customized models used in price forecasting were established during the evolution of the e-pricing system, and they are now an indispensable component of Astellas Pharma EMEA's global pricing management. Based on the success of e-pricing in the EMEA affiliates, a global pricing system, which includes the Americas and the Asia-Pacific regions, was rolled-out in October 2014. The global system is being evolved based on functionality already available within the e-pricing system currently used within the EMEA region.

## Summary and conclusion

In a rapidly changing regulatory environment, where countries increasingly look to each other for direction on pharmaceutical pricing and reimbursement, the effects of reference pricing influence pharmaceutical industry sustainability [16]. Price optimization is a highly complex procedure, particularly for global corporations, and rapid and accurate pricing decisions are linked with optimal revenue flows. Global corporations use computer-based pricing systems to support pricing decisions across a range of industries [17]. The complexity of the pharmaceutical market makes the use of such systems crucial to inform launch and pricing decisions and to help manage prices throughout the product lifecycle.

Although a number of business applications are available from third-party suppliers that achieve similar data management and reporting solutions, Astellas Pharma EMEA considered that development of a tailored system was more appropriate to their in-house processes. The Astellas e-pricing system has improved the efficiency, reliability, compliance, transparency and ease of access to drug pricing, approval and reimbursement information within Astellas Pharma EMEA. In addition, it has significantly enhanced reporting capabilities and the ability to analyse data easily. As a result, affiliates no longer make decisions without taking into consideration the wider impact on the performance of the business. The most important limitations of the system are related to the quality of the information contained within it; key variables may include the efficiency of implementing pricing and reimbursement data changes into the system, and the proficiency of quality control measures.

In conclusion, the ability to model the financial impact of a specific price level accurately is crucial for a product’s viability. The ability to do this relies on the reliability of the available price-related data. Electronic pricing information management systems are essential for any multinational pharmaceutical company. The development of a bespoke e-pricing system enabled Astellas Pharma EMEA to take full ownership of pricing considerations and to improve the decision-making process.

## References

[1] OECD Health policy studies. Pharmaceutical pricing policies in a global market. Paris: OECD Publications; 2008 Available from: http://www.keepeek.com/Digital-Asset-Management/oecd/social-issues-migration-health/pharmaceutical-pricing-policies-in-a-global-market_9789264044159-en#page1

[2] EvaluatePharma® World preview 2016, Outlook to 2022. 9th ed. 2016 9 Available from: http://info.evaluategroup.com/rs/607-YGS-364/images/wp16.pdf

[3] Danzon PM, Towse A, Mulcahy AW. Setting cost-effectiveness thresholds as a means to achieve appropriate drug prices in rich and poor countries. Health Aff (Millwood). 2011;30(8):1529–7. DOI:10.1377/hlthaff.2010.0902 21821570

[4] Kanavos P, Vandoros S, Irwin R, et al Differences in costs of and access to pharmaceutical products in the EU. Brussels: European Parliament; 2011 Available from: http://www.europarl.europa.eu/document/activities/cont/201201/20120130ATT36575/20120130ATT36575EN.pdf

[5] Espin J, Rovira J, De Labry AO WHO/HAI project on medicine prices and availability-Working paper 1: external reference pricing. World Health Organization and Health Action International 2011. Available from: http://www.haiweb.org/medicineprices/24072012/ERPfinalMay2011.pdf

[6] Carone G, Schwierz C, Xavier A Cost-containment policies in public pharmaceutical spending in the EU. European Commission (Directorate General for Economic and Financial Affairs) European Economy. Brussels: Economic Papers 461; 2012 DOI:10.2765/27111.

[7] Vogler S, Zimmermann N, Leopold C, et al Pharmaceutical policies in European countries in response to the global financial crisis. South Med Rev. 2011;4(2):69–79. Available from: http://apps.who.int/medicinedocs/documents/s19046en/s19046en.pdf 23093885 10.5655/smr.v4i2.1004 PMC3471176

[8] Leopold C, Vogler S, Mantel-Teeuwisse AK, et al Differences in external price referencing in Europe: a descriptive overview. Health Policy. 2012;104(1):50–60. DOI:10.1016/j.healthpol.2011.09.008 22014843

[9] Business Monitor International The worldwide guide of pricing and reimbursement. Espicom. 2015 7 London (UK).

[10] “Revenue Leakage – Pharma’s $11 Billion Problem”, Health Industry Insights, December 2009. [Reported in: Enterprise Revenue Dynamics For Pharmaceutical Manufacturers. The Integration of Contracts, Pricing, and Compliance for Better Performance Outcomes. Revitas White Paper. 2012 7 Available from: http://www.revitasinc.com/images/uploads/whitepapers/WP_006_ERD_for_Pharma.pdf

[11] Accenture Going for Growth: Balancing Price and Cost in a Recovering Global Economy. [Reported in: Homer P. Maximize global revenue from pharmaceutical product launches. A shot in the arm. SAS Health and Life Sciences blog. Available from: http://blogs.sas.com/content/hls/2015/10/23/maximize-global-revenue-from-pharmaceutical-product-launches/

[12] Robinson J Pricing management: it’s all about the data. Pharmaceutical executive [cited 2014 2 21]. Available from: http://www.pharmexec.com/pricing-management-its-all-about-data

[13] Royce WR Managing the development of large software systems Proceedings of The Institute of Electrical and Electronics Engineers (IEEE) Western Electronic Show and Convention (WESCON), 1970 Aug. 1–9 Available from: http://www.cs.umd.edu/class/spring2003/cmsc838p/Process/waterfall.pdf

[14] Waterfall Development Methodology Available from: http://learnaccessvba.com/application_development/waterfall_method.htm

[15] Centers for Medicare & Medicaid Services (CMS) Office of Information Service (2008). Selecting a development approach. USA Department of Health and Human Services (HHS). Available from: https://www.cms.gov/research-statistics-data-and-systems/cms-information-technology/xlc/downloads/selectingdevelopmentapproach.pdf

[16] Toumi M, Rémuzat C, Vataire A-L, et al External reference pricing of medicinal products: simulation-based considerations for cross-country coordination. European Commission. 2013 12 Availabe from: http://ec.europa.eu/health/healthcare/docs/erp_reimbursement_medicinal_products_en.pdf

[17] Bright JK, Kiewell D, Kincheloe AH Pricing in a proliferating world. The McKinsey Quarterly. Profiting from Proliferation. 2006 8 73–83. Available from: http://www.mckinsey.com/business-functions/marketing-and-sales/our-insights/pricing-in-a-proliferating-world

